# Metformin and gonadotropins for ovulation induction in patients with polycystic ovary syndrome: a systematic review with meta-analysis of randomized controlled trials

**DOI:** 10.1186/1477-7827-12-3

**Published:** 2014-01-03

**Authors:** Stefano Palomba, Angela Falbo, Giovanni B La Sala

**Affiliations:** 1 Obstetrics and Gynecology Unit, Department of Obstetrics, Gynecology and Pediatrics, Azienda Ospedaliera ASMN, Istituto di Ricovero e Cura a Carattere Scientifico, Viale Risorgimento 80, 42123 Reggio Emilia, Italy; 2 University of Modena and Reggio Emilia, Via Università 4, 41100 Modena, Italy

**Keywords:** Gonadotropins, Infertility, Meta-analysis, Metformin, PCOS, RCT

## Abstract

The current systematic review with meta-analysis of randomized controlled trials (RCTs) was aimed to evaluate the effects of metformin on reproductive outcomes in patients with polycystic ovary syndrome (PCOS) who receive gonadotropins for ovulation induction. After systematic review of electronic databases and websites for registration of RCTs, a total of 7 RCTs reporting data on 1023 cycles were included in the final analysis. Descriptive data showed an overall low studies’ quality due to unclear sequence generation and allocation concealment, lack of blinding procedure, incomplete outcome data and several biases and/or confounders. Data synthesis showed that metformin improved live-birth (odds ratio [OR] = 1.94, 95% confidence interval [CI] 1.10 to 3.44; *P* = 0.020) and pregnancy (OR = 2.25, 95% CI 1.50 to 3.38; *P <* 0.0001) rates, without significant heterogeneity across the studies (*P* = 0.230, estimation of inconsistency = 30%; and *P* = 0.710, estimation of inconsistency = 0%, respectively, for live-birth and pregnancy rates). A significant reduction of cancellation rate was observed after metformin administration (OR = 0.41, 95% CI 0.24 to 0.72, *P* = 0.002) without significant heterogeneity across the studies (*P* = 0.500, estimation of inconsistency = 0%). Metformin administration influenced or did not influence other secondary endpoints assessed with a significant heterogeneity. In conclusion, metformin administration increases the live-birth and pregnancy rate in PCOS patients who receive gonadotropins for ovulation induction. Further well designed, blinded, placebo-controlled, and adequately powered RCTs are need to confirm that metanalytic results.

## Background

Metformin, an insulin sensitizer widely used for treating type-2 diabetes mellitus, is employed in patients with polycystic ovary syndrome (PCOS) in light of the scientific data showing the pivotal role of insulin resistance in the pathogenesis of the syndrome, and of its beneficial effects on metabolism and ovulatory function in PCOS women [1].

Experimental and translational data seem to suggest that metformin could influence the ovarian response to gonadotropins. In fact, it improves not only the systemic insulin sensitivity and serum androgen levels in PCOS patients [1,2] but also their ovarian morphology [3] and environment [2] by improving the intra-ovarian hyperandrogenism through local effect on ovarian steroidogenesis [4] and the intra-ovarian insulin-resistance [2,3] interfering with autocrine/paracrine insulin-related signaling [5,6]. These actions on the peripheral tissues are irrespective of systemic improvement in metabolism and ovulatory function [2]. In addition, scientific data suggest an improvement of the endometrial receptivity in PCOS patients under metformin treatment [7,8].

Gonadotropin administration represents a widely accepted therapeutic option to induce ovulation in PCOS patients with anovulatory infertility [9,10], despite its high direct and indirect costs and its high risk of side effects.

Based on these considerations, metformin theoretically could induce a normalization of the abnormal ovarian responsiveness to gonadotropins, which is characteristic in PCOS patients, as well as lead to an improvement of their endometrial receptivity with an overall beneficial effect in terms of pregnancies and live births. Furthermore, a previous meta-analysis [11] published on 2006 demonstrated that data regarding metformin administration during gonadotropin ovulation induction were inconclusive. In fact, the inclusion of only two randomized controlled trials (RCTs) with less than 50 subjects each and the lack of studies aimed to assess the pregnancy rates limited the power of the analysis to exclude a treatment benefit [11].

The current study was aimed to clarify the effects of metformin in infertile PCOS patients who receive gonadotropin for ovulation induction through a systematic review with meta-analysis of available RCTs.

## Methods

The protocol design followed the Preferred Reporting Items for Systematic Reviews and Meta-Analyses (PRISMA) guidelines for reporting systematic reviews and meta-analyses of RCTs [12].

### Study selection

Criteria for inclusion and exclusion of studies were established prior to the literature search.

Only RCTs characterized by symmetric interventions between the two treatment arms, i.e., patients who received the same protocol for ovulation induction with gonadotropins and then randomized to metformin or to placebo/no treatment, were included. Crossover studies were also included, although only data from the pre-crossover phase were considered for meta-analysis.

Studies were excluded if non-randomized; if any follow-up data were either not available, not extractable, not documented, or if the authors did not respond; if data were inconsistent or suspected duplicate (corresponding author was contacted by email and asked for clarification, and, if no clarification was obtained, data sets were considered overlapping and only the wider ones were included); if they included subjects who received gonadotropins for in vitro fertilization (IVF) programs.

No limit was given for PCOS diagnosis, dose and protocol of intervention proposed, type of gonadotropin used, and/or stimulation protocol employed.

### Search strategy

The bibliographic search for identification of articles, abstracts, and study protocols was conducted monthly up to October 2013, with no language restriction.

A combination of the following medical subject headings or keywords was included: “controlled ovarian hyperstimulation”, “controlled ovarian stimulation”, “fertility”, “gonadotrophins”, “gonadotropins”, “infertility”, “insulin sensitisers”, “insulin-sensitising drugs”, “insulin sensitizers”, “insulin-sensitizing drugs”, “live-birth”, “metformin”, “OHSS” “ovarian hyperstimulation syndrome”, “ovulation induction”, “PCOS”, “polycystic ovarian disease”, “polycystic ovary disease”, “polycystic ovary syndrome”, “polycystic ovarian syndrome”, “pregnancy”, “randomised controlled trials”, “randomized controlled trials”, “RCTs”, “sterility”, “sub-fertility”.

The following data sources were electronically searched: MEDLINE through PubMed (1966 to October 2013), EMBASE (1966 to September 2013), CINAHL (1981 to October 2013), Cochrane Library (1970 to October 2013), Clinical Evidence, UpToDate, and DARE for relevant studies. The Institute for Scientific Information (ISI), Web of Science, Scopus, Google Scholar, and the websites for the registration of controlled trials were also consulted for relevant clinical trials up to October 2013.

The bibliographies of retrieved articles, books and expert opinion review articles were manually searched and reviewed. No systematic attempt to search the grey literature, defined as information produced on all levels of government, academics, and/or business and industry in electronic and print formats not controlled by commercial publishing (International Conferences on grey literature, New York 2004), was made.

First, the titles and abstracts were screened and potentially relevant articles were identified and reviewed for inclusion/exclusion criteria. Then, the protocols and results of the studies were examined according to specific inclusion criteria. Lastly, only studies that met the inclusion criteria were considered for the final analysis.

Two independent reviewers (A.F., S.P.) not blinded at any point to the authors or sources of publication simultaneously reviewed the full manuscripts of all citations that possibly matched the predefined selection criteria. Final inclusion or exclusion decisions were made on examination of the full manuscripts. Disagreements between the reviewers on inclusion were discussed and solved by consensus or arbitration after consultation with an independent third author (G.B.L.S.).

### Data extraction

The primary endpoints were the live-birth rate, defined as the number of deliveries that resulted in at least one live born baby for initiated cycle, and the pregnancy rate, defined as number of pregnancies per initiated cycles [13].

The secondary endpoints included the rates of miscarriages, multiple pregnancy, cancelled cycles for either poor- or hyper-response, ovarian hyperstimulation syndrome (OHSS), the stimulation length, the gonadotropin dose, and the serum estradiol (E_2_) levels at human chorionic gonadotropin (hCG) injection [13].

The process of data abstraction examined the methodological and procedural characteristics of each study as well as a wide range of variables, including demographic, hormonal and metabolic characteristics of the study population, definition of PCOS, and treatments received with particular regard for type, protocols, and doses. These data were all extracted and tabulated.

The collaboration of all corresponding authors was requested, whenever possible, to obtain data missing from the papers included in the study, as well as unpublished and preliminary data.

### Quantitative data analysis and synthesis

Statistical analyses were performed according to the statistical guidelines for review authors developed by The Cochrane Collaboration and published in the Cochrane Handbook for Systematic Reviews of Interventions [14]. All the statistical analyses were performed by using Review Manager Version 5 [15], provided by the Cochrane Menstrual Disorders and Subfertility Group.

The analysis of the treatment effect was performed according to the intention-to-treat (ITT) principle considering dropouts and missing data as treatment failures. In consideration of the potential effect of metformin in a pretreatment phase on pregnancy and live-birth, when possible, the analysis was also performed also according to the per-protocol method, considering the results only from the patients who really received the infertility treatment (gonadotropins with and/or without metformin).

Odds ratio (OR), with 95% confidence interval, was used as a valid way of describing an intervention effect for each dichotomous outcome using the Mantel-Haenszel method [16]. In particular, OR describes the multiplication of the odds of the outcome that occur with use of the intervention [16]. Continuous outcome differences between the two groups were presented as mean difference (MD) with 95% confidence interval.

A fixed-effect model was initially employed in the analysis, unless a significant heterogeneity occurred; a random effects model analysis was used in order to account for the extra uncertainty due to heterogeneity.

Potential heterogeneity of the treatment effects of each trial was examined by testing for interactions between source trial and treatment effects and estimation of inconsistency (*I*^*2*^) [14,17]. Specifically, *I*^*2*^ represents an estimate of the degree of inconsistency among studies; *I*^*2*^ scores from 0% to 40% might not be important; from 30% to 60% may represent moderate heterogeneity; from 50% to 90% may represent substantial heterogeneity; and from 75% to 100% considerable heterogeneity [14].

A *P* value lower than 0.05 or 95% CI that did not contain unity was considered statistically significant. A statistical trend was arbitrarily established for *P* values that ranged between 0.05 and 0.09.

The number needed to treat (NNT) was calculated only for outcomes which were statistically significantly different between metformin and control/placebo groups, i.e. the expected number of people who need to receive the experimental (metformin) rather than the comparator intervention (no metformin) for one additional person to either incur or avoid an event in a given time frame.

## Results

### Search data

**Figure 1:**
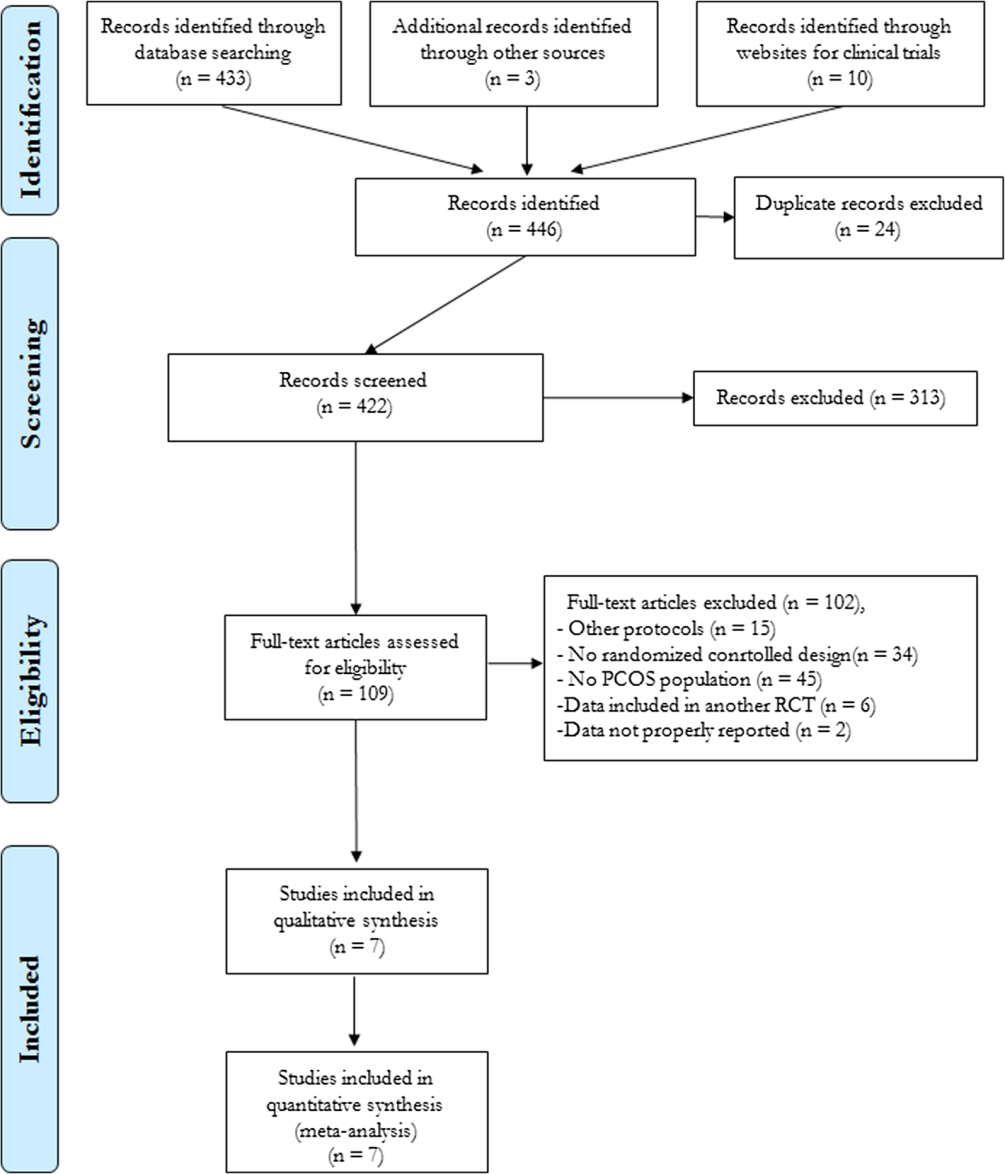
Flow diagram of the study selection.

Figure 1 shows the flow diagram of the study selection according to the PRISMA statement [12].

No unpublished study or in-progress study protocol was identified.

Seven studies [18-24] were included in the final analysis.

### Studies description

The included studies [18-24] reported data on an overall population of 334 PCOS subjects (167 and 167 for metformin and control arm, respectively). A total of 1023 cycles were analyzed (438 and 585 cycles under metformin and no metformin, respectively).

**Figure 2:**
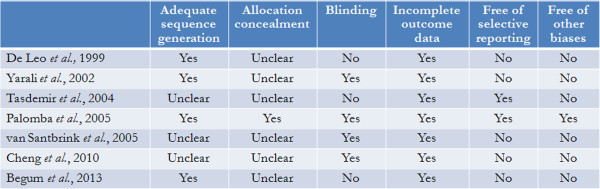
Study quality assessment.

Study quality is detailed in Figure 2. An overall low studies’ quality due to unclear sequence generation and allocation concealment, lack of blinding procedure, incomplete outcome data and several biases and/or confounders were observed.

Table 1 summarizes the main characteristics of the RCTs included in the final analysis.

**Table 1:** Characteristics of included studies

	**Participants**	**Outcomes**	**Notes**
**De Leo *et al *** [18]	*Sample size:*	- N. of ampoules of huFSH.	Crossover study.
	20 subjects	- Serum E_2_ levels.	No data on live-births.
	*Exclusion:*	- Days of treatment.	No blind design.
	- Abnormal FSH and/or prolactin levels.	- Cancelled cycles.	No criteria for defining OHSS.
	- Abnormal thyroid function.	- Incidence of OHSS.	No criteria for cycle cancellation.
	- Congenital adrenal hyperplasia.	- Pregnancy rate.	
	- Abnormal partner’s seminal parameters.	- Side effects.	
	- Drug assumption 2 mths prior to the study.		
**Yarali *et al *** [19]	*Sample size:*	- N. of ampoules of rFSH.	Data of spontaneous ovulation during the pre-treatment phase were excluded.
	32 subjects	- Serum E_2_ levels.	
	*Exclusion:*	- Days of treatment.	No criteria for defining OHSS.
	- Any infertility factor other than PCOS (by semen analysis, hysterosalpingography, and/or laparoscopy).	- Cancelled cycles.	No data on OHSS.
	- Endocrinopathies.	- Pregnancy rate.	No data on live-births.
	- Abnormal glucose tolerance, IGT or type-2 DM.	- Endometrial thickness.	Cycle was cancelled in presence of more than 3 follicles ≥15 mm, or in absence of ovarian response after 35 days of treatment.
	- Use of medications known to alter insulin secretion or action.	- Side effects.	
**Tasdemir *et al *** [20]	*Sample size:*	- rFSH IU.	No blind design.
	32 subjects	- serum E_2_ levels.	No criteria for cycle cancellation.
	*Exclusion:*	- N. dominant follicles.	No criteria for defining OHSS.
	- Age <20 > 34.	- Days of treatment.	No data on live-births.
	- Congenital adrenal hyperplasia.	- Endometrial thickness.	Not specified n. of side effects.
	- Hyperprolactinemia.	- Cancelled cycles.	
	- Hypothyroidism.	- Incidence of OHSS.	
	- Abnormal renal and liver tests.	- Pregnancy rate.	
	- Use of drugs with possible effect on endogenous sex hormones.	- Multiple pregnancies.	
	- Type 1-2 DM.	- Side effects.	
	- Hypophysal insufficiency.		
	- Any infertility factor other than PCOS (by semen analysis, hysterosalpingography, and/or laparoscopy).		
**Palomba *et al *** [21]	*Sample size:*	- N. of ampoules of hpFSH.	Only insulin-resistant women were included.
	70 subjects	- Serum E_2_ levels.	IUI was performed in ovulating women who failed to conceive.
	*Exclusion:*	- N. dominant follicles.	TI was performed in non-ovulating women.
	- Age <20 or >34 years.	- Days of treatment.	Cycle was cancelled in presence of more than 3 follicles ≥14 mm, or in absence of ovarian response after 35 days of treatment.
	- BMI >30 and <18 kg/m^2^.	- Cancelled cycles.	
	- Neoplastic, metabolic (including glucose intolerance), hepatic, and cardiovascular disorder or other concurrent medical illness.	- Incidence of OHSS.	
	- Hypothyroidism.	- Ovulation rate.	
	- Hyperprolactinaemia.	- Rate of mono-ovulatory cycles.	
	- Cushing’s syndrome; non-classical congenital adrenal	- Pregnancy rate.	
	- hyperplasia.	- Multiple pregnancy rate (primary end-point).	
	- Abuse of alcohol.	- Abortion.	
	- Current or previous (within 6 mths) use of oral contraceptives, glucocorticoids, antiandrogens, antidiabetic, and anti-obesity and hormonal drugs.	- Live-birth rate.	
	- Organic pelvic diseases.	- Side effects.	
	- Previous pelvic surgery.		
	- Suspected peritoneal factor infertility.		
	- Tubal or male factor infertility (by hysterosalpingogram and semen analysis).		
	- Intention to start a diet or a specific program of physical activity.		
**van Santbrink *et al *** [22]	*Sample size:*	- Units of rFSH (primary end-point).	Only insulin-resistant women were included.
	20 subjects	- Serum E_2_ levels.	No clear definition for CC-resistance and CC-failure.
	*Exclusion:*	- Days of treatment (primary end-point).	
	- Age ≤18 ≥ 37 yrs	- Cancelled cycles.	No specific definition for PCOS.
	- Abnormal serum E_2_ and FSH levels.	- Incidence of OHSS.	No criteria for defining OHSS.
	- Abnormal serum prolactin and thyroxine levels.	- Ovulation rate.	No specification of the time of metformin and placebo administration.
	- DM.	- Rate of mono-ovulatory cycles.	
	- Signs of liver or kidney insufficiency and heart or vascular disease.	- Pregnancy rate.	The study was divided into two phases. Only the 2^nd^ phase was considered in the analysis.
		- Multiple pregnancy rate.	
		- Abortion.	No data on live-births.
		- Serious side effects.	Cycle was cancelled in presence of more than 3 follicles ≥15 mm, or in absence of ovarian response at the maximum dosage (225IU rFSH daily).
**Cheng *et al *** [23]	*Sample size:*	- N. of ampoules of HMG.	No data on the days of treatment.
	60 subjects	- Serum E_2_ levels.	No data of the multiple pregnancies.
	*Exclusion:*	- N. dominant follicles.	No data on live-births.
	- Age ≥ 40 yrs.	- Cancelled cycles.	No data on side effects.
	- Endometrial pathology.	- Incidence of OHSS.	No criteria for defining OHSS.
	- Abnormal glucose tolerance (75g OGTT).	- Ovulation rate.	Cycle was cancelled in presence of more than 4 dominant follicles.
	- Any infertility factor other than PCOS.	- Rate of mono-ovulatory cycles.	
	- Other common causes of hyperandrogenism.	- Pregnancy rate (primary end-point).	
	- Prolactinoma.		
	- Congenital adrenal hyperplasia.		
	- Cushing syndrome.		
	- Virilizing ovarian or adrenal tumours.		
	- Hormonal drugs assumption 3 mths prior to the study.		
**Begum *et al *** [24]	*Sample size:*	- Ovulation rate.	No blind design.
	110 subjects	- Miscarriage.	No criteria for defining OHSS.
	*Exclusion:*	- Perinatal outcome.	No criteria for cycle cancellation due to hyper-response.
	-DM.	- Pregnancy rate (primary end-point).	
	- Altered glucose metabolism.	- Live-birth rate (primary end-point).	No clear strategy (TI or IUI).
	- Hyperprolactinemia.		
	- Hypothyroidism.		
	- Endometriosis.		
	- Pelvic inflammatory disease.		
	- Tubal factor infertility.		
	- Partner abnormal semen parameters.		

BMI: body mass index; CC: clomiphene citrate; DM: diabetes mellitus; E_2_: estradiol; GIR: glucose to insulin ratio; HMG: human menopausal gonadotropins; hpFSH: human purified follicle-stimulating hormone; huFSH: human urinary FSH; IGT: impaired glucose tolerance; IUI: intrauterine insemination; LH: luteinizing hormone; OGTT: oral glucose tolerance test; OHSS: ovarian hyper-stimulation syndrome; PCOS: polycystic ovary syndrome; rFSH: recombinant FSH; SHBG: sex-hormone binding globulin; TI: timed intercourse.

A wide variability was found across studies in the characteristics of participants, interventions performed and outcomes measured.

In 2 [23,24] and 3 [18,19,21] RCTs, the diagnosis of PCOS was made according to the European Society of Human Reproduction and Embryology (ESHRE)/American Society of Reproductive Medicine (ASRM) [25] or to the National Institute of Health (NIH) [26] criteria, respectively, whereas in 2 RCTs [20,22] non-standardized criteria were used. In particular, PCOS was diagnosed by the presence of oligomenorrhea, clinical and biochemical signs of hyperandrogenism, polycystic ovaries (PCO) and follicle stimulating hormone (FSH)/luteinizing hormone (LH) level higher than 2 in 1 RCT [20], whereas it was diagnosed by the presence of oligomenorrhea (an interval of at least 56 days between menses) or amenorrhea (an interval of at least 6 months between menses) in the other one RCT [22].

PCOS phenotype of the studied population was defined in no RCT.

In 4 studies [18-20,24], clomiphene citrate (CC)-resistance was a specific inclusion criterion, whereas 3 other RCTs [21-23] included patients with CC-resistance or CC-failure.

Two RCTs [19,23] included only women with normal glucose tolerance. On the other hand, in 2 other RCTs [21,22], the presence of insulin resistance, univocally defined as a glucose-to-insulin ratio <4.5 mg/10-4, was a specific inclusion criterion.

In 5 RCTs, a chronic [21,22] or a traditional low-dose step-up [18-20] protocol was used with a starting dose of 75 IU [18-21] or 50 IU [22] of recombinant FSH (rFSH) [19,20,22], human urinary FSH (huFSH) [18] or highly purified FSH (hpFSH) [21]. In 1 study [24], an alternate day protocol was used with a starting dose of 75 IU rFSH. In another study [23] a fixed dose protocol consisting in 50 mg daily of CC from days 3 to 5 of the menstrual cycle plus human menopausal gonadotropins (HMG) administrated from day 5 with a starting dose of 75 IU daily was used.

Metformin was administered as pretreatment in all studies with the exception of 1 RCT [23] with a daily dose of 1500 mg [18,23,24] or 1700 mg [19-22]. Pretreatment duration was extremely variable, i.e., 4 [18,24], 6 [19], 8 [20], 12 [21], or 14 [22] weeks before gonadotropin administration. Metformin use continued until ovulation triggering in 3 RCTs [18,19,22] or pregnancy test in 4 RCTs [20,21,23,24], whereas in no case was treatment continued during pregnancy.

The control group received gonadotropins plus placebo in 4 RCTs [19,21,22,24] or gonadotropins alone in 3 RCTs [18,20,24].

In all studies [18,24], ovarian maturation was triggered by means of urinary hCG at the dosage of 10,000 IU [18-21,24] or 5,000 IU [22,23], when at least 1 periovulatory follicle was detected [18,23].

Criteria initially adopted for cycle cancellation were heterogeneous among RCTs [18-24]. In particular, cycle was cancelled due to hyper-response in presence of more than 3 [18,19,21,22], 4 [23], or 5 [20] periovulatory follicles. No criteria for cycle cancellation due to hyper-response was given by Begum et al. [24]. Criteria for cycle cancellation due to hypo-response were not reported in 3 RCTs [18,20,23]; instead, 3 RCTs [19,21,24] reported their cycle cancellation criterion as being no follicular response after 30 [24] or 35 [19,21] days of stimulation and 1 RCT [22] in case of no follicular response with a maximum dosage of 225IU rFSH daily. At study end, in 5 RCTs [18,20-23] cycles were always cancelled for excessive ovarian response, whereas in only 2 RCTs [19,24] the cycles were cancelled for no ovarian response.

Timed intercourses were performed in 5 RCTs [18-20,22,23], except in 1 RCT [21], in which women who previously failed to ovulate underwent timed intercourse whereas women who ovulated in the previous cycles but did not achieve a pregnancy underwent intrauterine insemination. In 1 study [24], the strategy used was not specified.

Luteal phase support was administrated in only 1 RCT [23]. It consisted in 20 mg/day progesterone cream given topically for 14 days after ovulation triggering [23].

### Meta-analysis

#### ITT analysis

**Figure 3:**
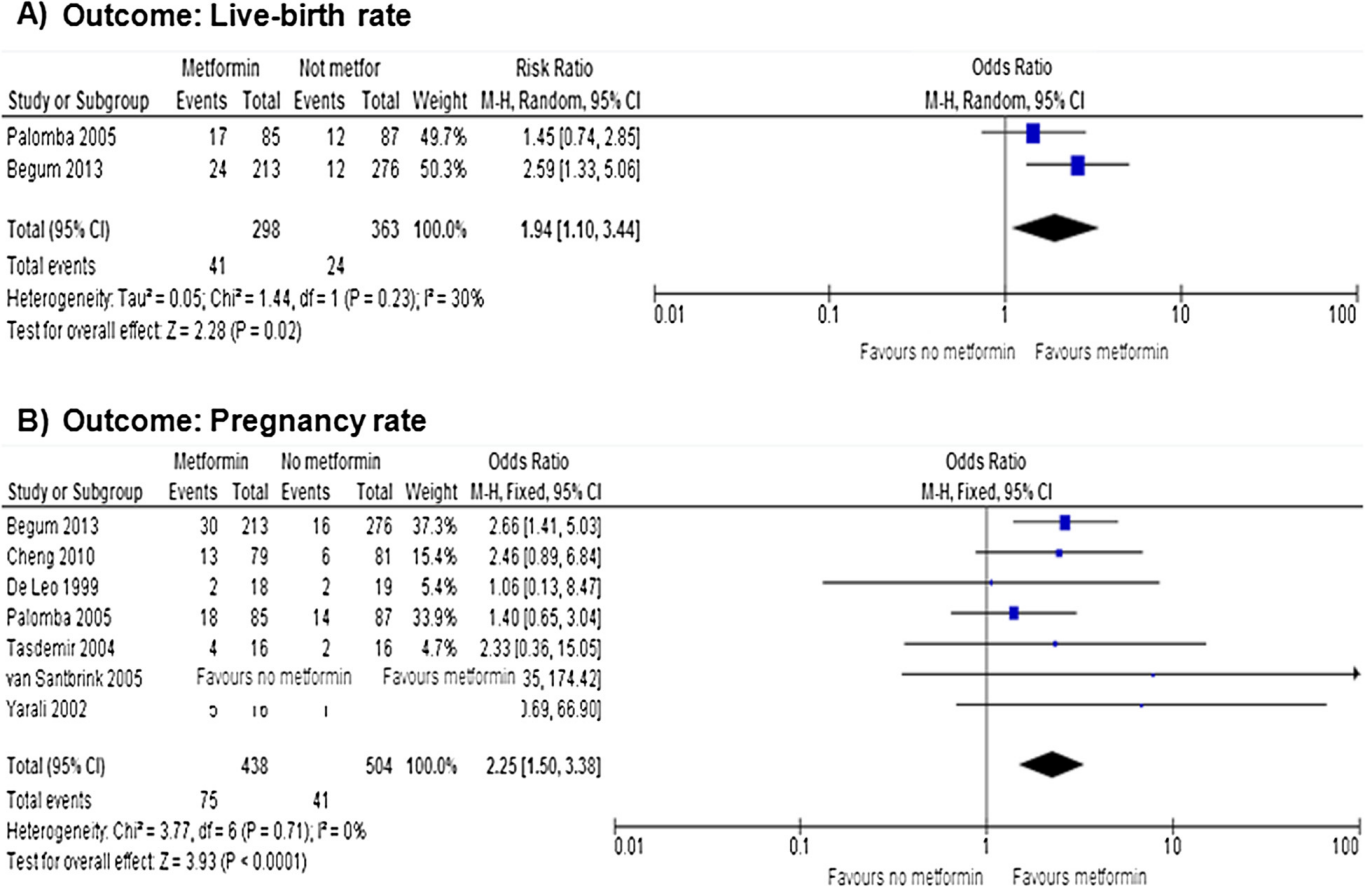
**Meta-analysis of primary endpoints performed using ITT principles.** Live-birth **(A)** and pregnancy **(B)** rates.

**Figure 4:**
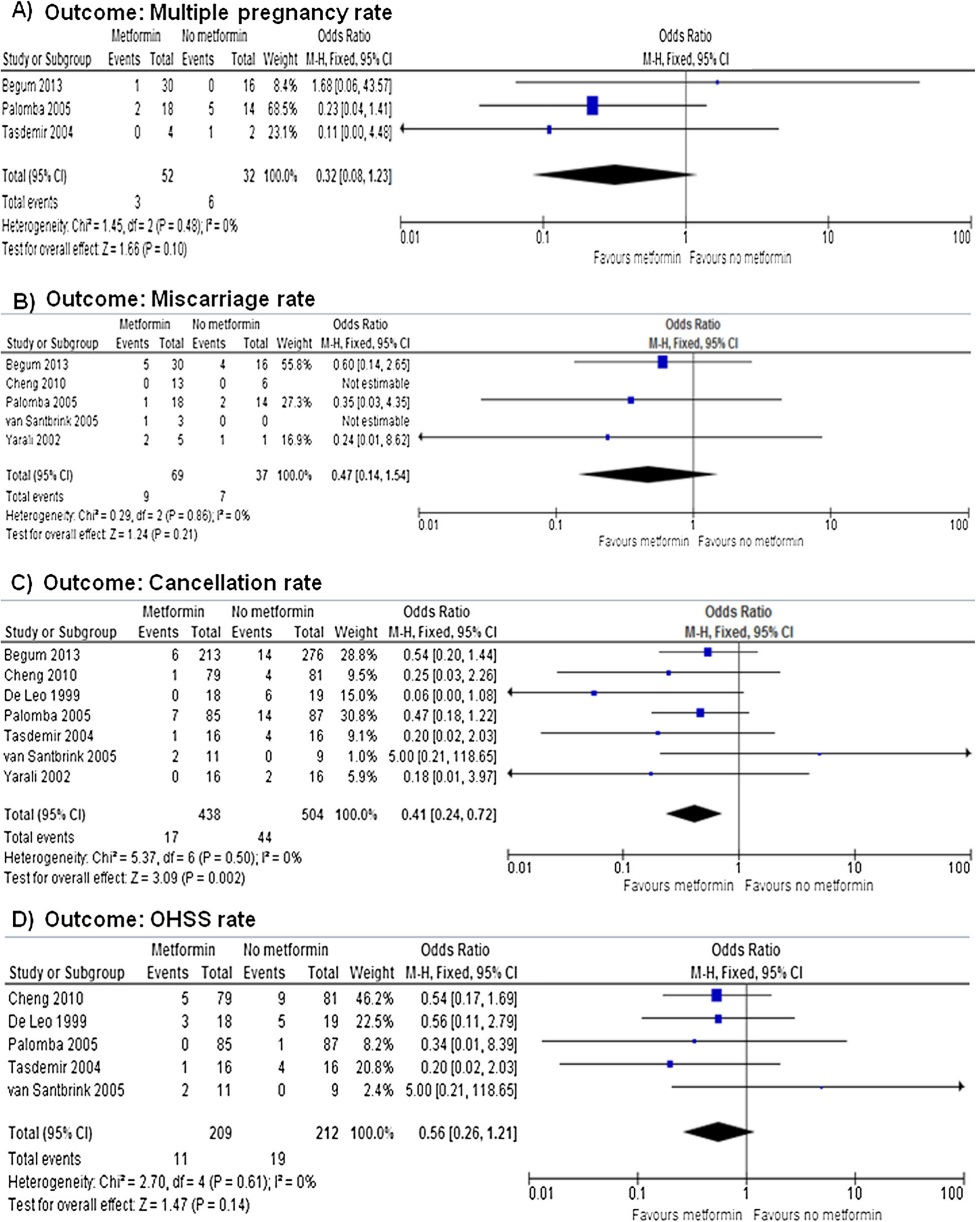
**Meta-analysis of clinical secondary endpoints performed using ITT principles.** Multiple pregnancy **(A)**, miscarriage **(B)**, cycle cancellation **(C)** and OHSS **(D)** rates.

**Figure 5:**
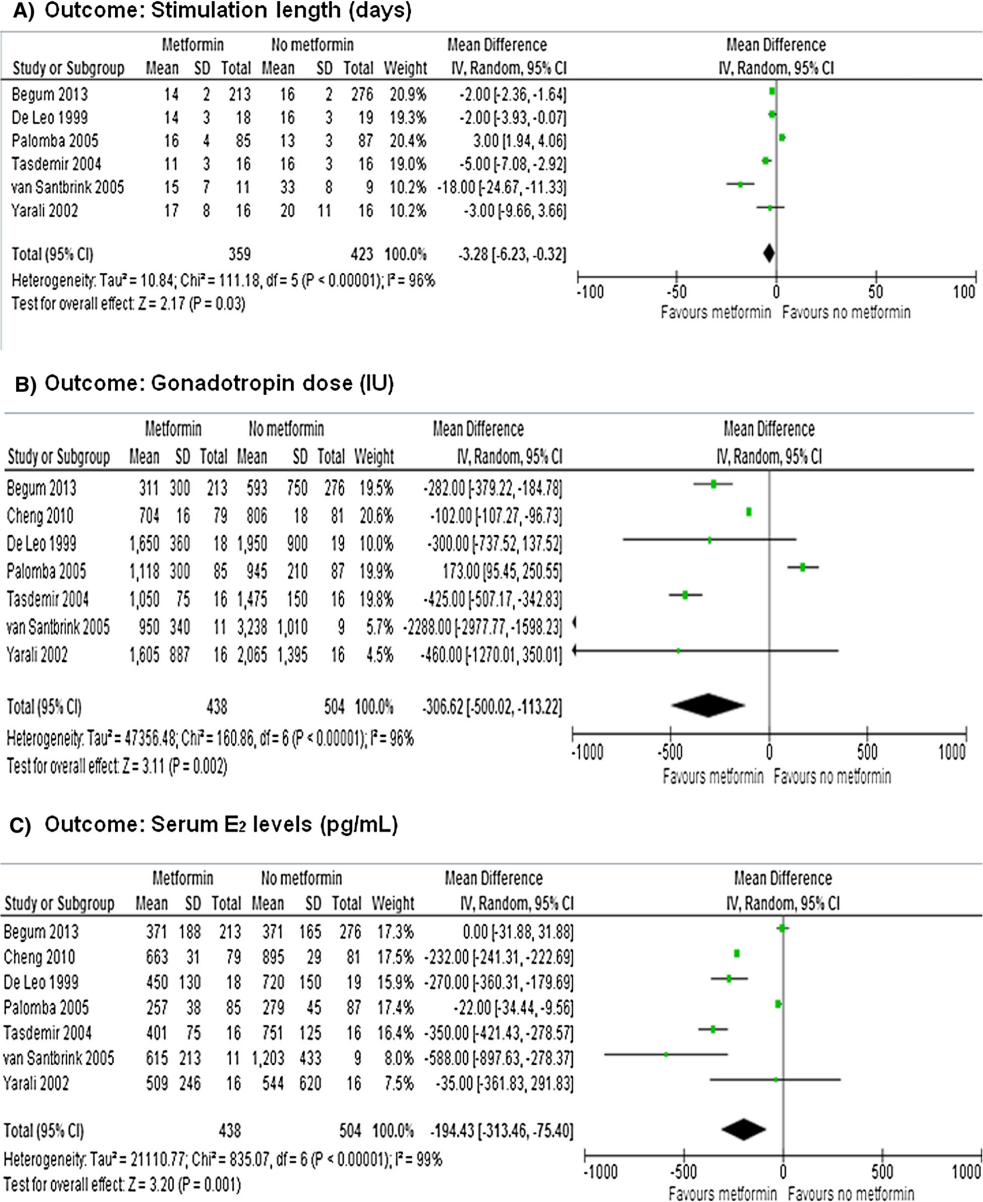
**Meta-analysis of stimulation secondary endpoints performed using ITT principles.** Stimulation lenght **(A)**, gonadotropin units **(B)** and serum E2 levels **(C)**.

Figures 3, 4 and 5 show the primary and secondary endpoints analyzed according to the ITT principle.

Data on the live birth rate were available in 2 RCTs [21,24]. Metformin increased significantly the live birth rate (OR = 1.94, 95% CI 1.10 to 3.44, *P* = 0.020), without significant heterogeneity across the studies (*P* = 0.230, *I*^2^ = 30%) (Figure 3A). Considering the live-birth as treatment-related event, the NNT was of 14 benefits (95% CI 8.5 to 40.4 benefits).

Based on meta-analysis of all 7 included RCTs [18-24], metformin significantly increased the pregnancy rate (OR = 2.25, 95% CI 1.50 to 3.38, *P <* 0.001), without significant heterogeneity across the studies (*P* = 0.710, *I*^2^ = 0%) (Figure 3B). Considering the pregnancy as treatment-related event, the NNT was of 5.7 benefits (95% CI 2 to 9 benefits).

Data on the rate of multiple pregnancies were formally reported in 3 RCTs [20,21,24]. After meta-analysis, no effect on multiple pregnancy rate was observed under metformin (OR = 0.32, 95% CI 0.08 to 1.23; *P* = 0.100), without significant heterogeneity across the studies (*P* = 0.480, *I*^2^ = 0%) (Figure 4A).

Data on the miscarriage rate were available in 5 RCTs [19,21-24]. After meta-analysis, no significant effect on the miscarriage rate was observed under metformin (OR = 0.47, 95% CI 0.14 to 1.54; *P* = 0.210), without significant heterogeneity across the studies (*P* = 0.290, *I*^2^ = 0%) (Figure 4B).

After meta-analysis of all included RCTs [18-24], a significant reduction of the cancellation rate was observed after metformin administration (OR = 0.41, 95% CI 0.24 to 0.72, *P* = 0.002), without significant heterogeneity across the studies (*P* = 0.500, *I*^2^ = 0%) (Figure 4C). Considering the reduction in cycle cancellation as treatment-related event, the NNT was of 25.6 benefits (95% CI 12 to 32 benefits).

Data on the OHSS rate were available in 5 RCTs [18,20-23]. After meta-analysis, no significant effect of metformin on the OHSS rate (OR = 0.56, 95% CI 0.26 to 1.21; *P =* 0.140) was observed, without significant heterogeneity across the studies (*P* = 0.610, *I*^2^ = 0%) (Figure 4D).

Data on the stimulation length were available in 6 RCTs [18-22,24]. After meta-analysis, a significant effect of metformin was observed on the stimulation length (MD = -3.28, 95% CI -6.23 to 0.32, *P* = 0.030), with significant heterogeneity across the studies (*P* < 0.0001, *I*^2^ = 96%) (Figure 5A).

After combining the data from all of the included RCTs [18-24], significantly less gonadotropin units were used under metformin (MD = -306.62, 95% CI -500.02 to -113.22, *P =* 0.002), with significant heterogeneity across the studies (*P* < 0.00001, *I*^2^ = 96%) (Figure 5B).

The meta-analysis of all included RCTs [18-24] showed a significant effect of metformin on serum E_2_ levels (MD = -194.43, 95% CI -313.46 to -75.40, *P =* 0.001), with significant heterogeneity across the studies (*P* < 0.00001, *I*^2^ = 99.0%) (Figure 5C).

### Per-protocol analysis

**Figure 6:**
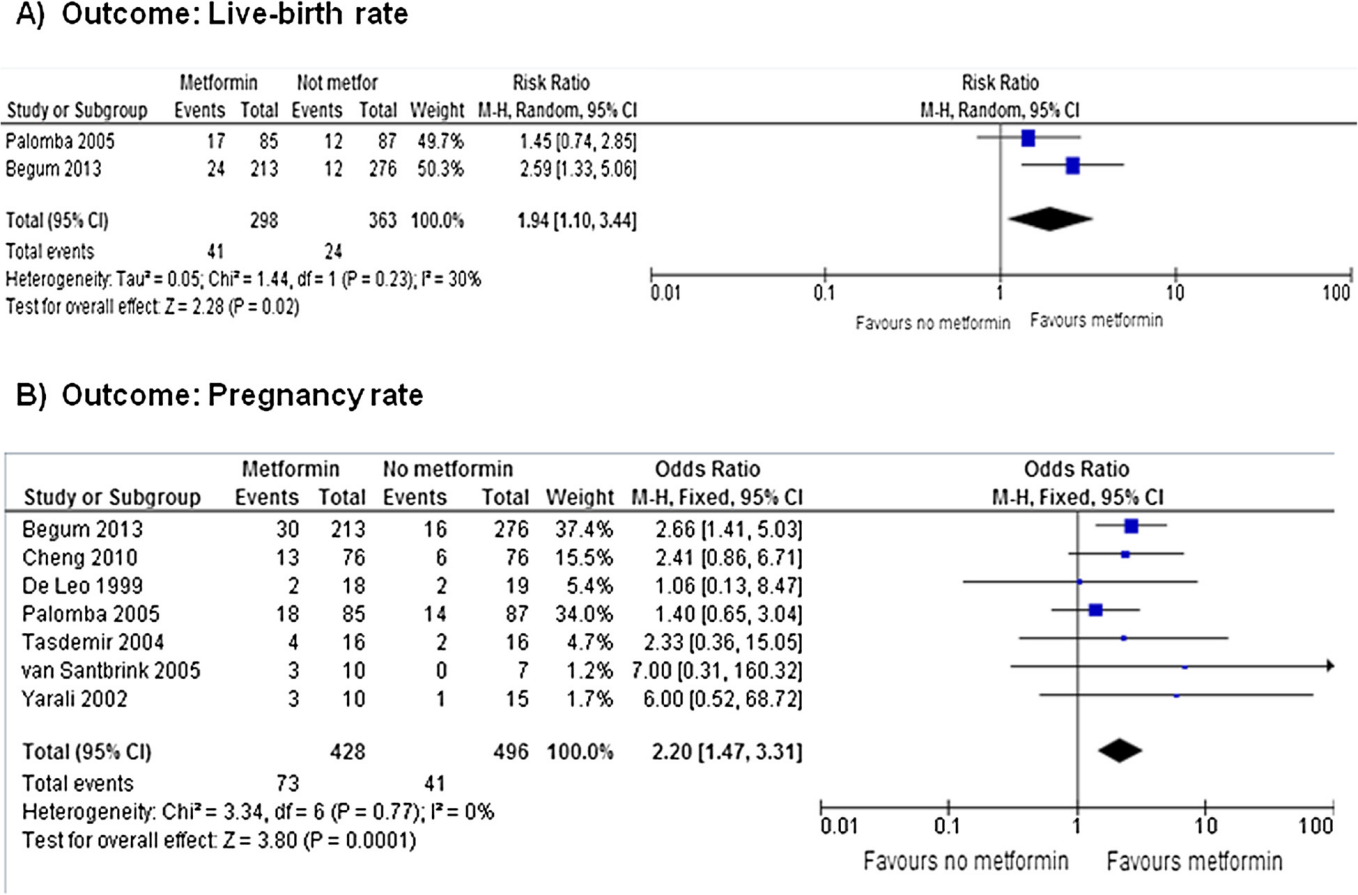
**Meta-analysis of primary endpoints performed using per-protocol principle.** Live-birth **(A)** and pregnancy **(B)** rates.

**Figure 7:**
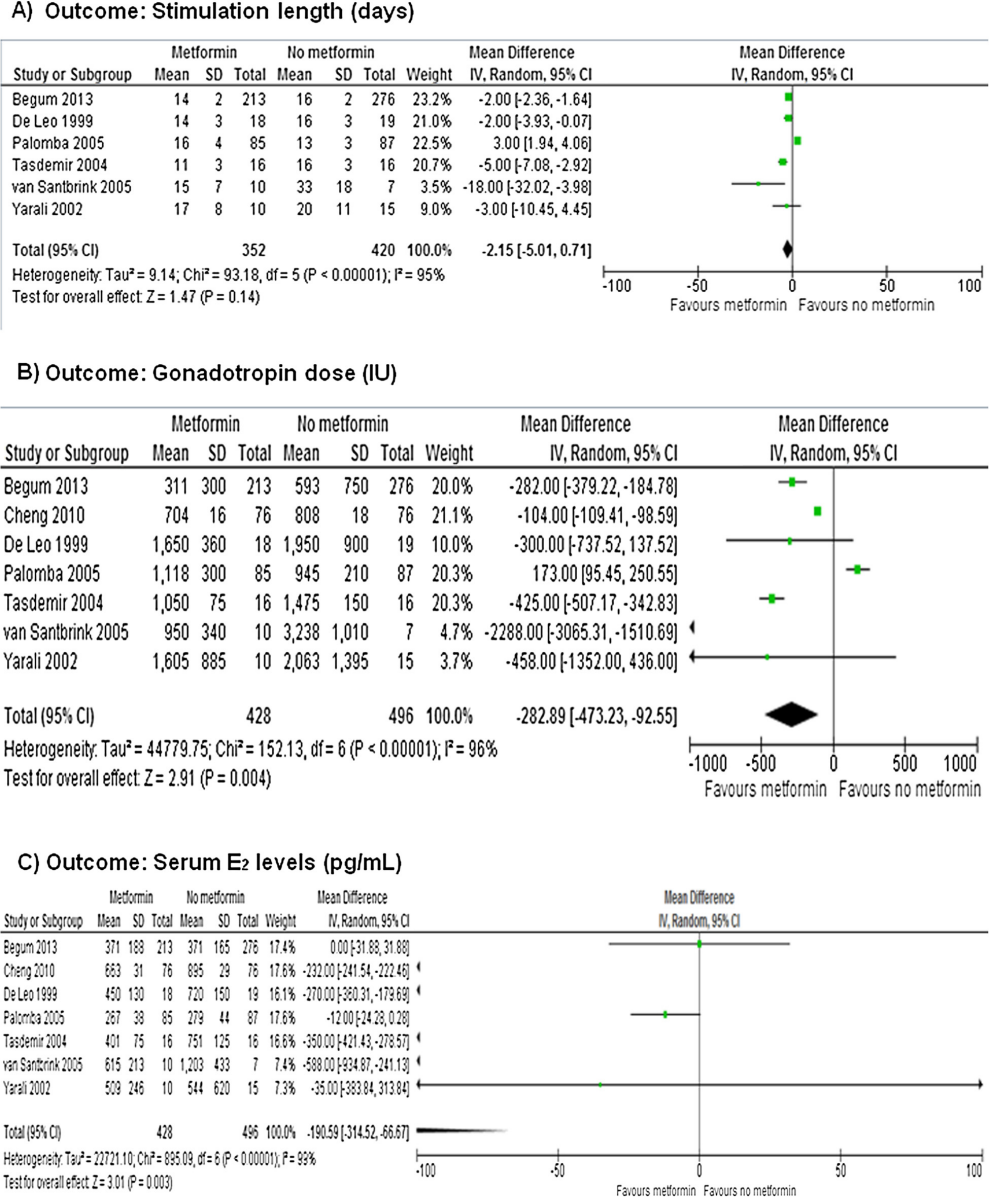
**Meta-analysis of secondary endpoints performed using per-protocol principle.** Stimulation lenght **(A)**, gonadotropin doses **(B)** and serum E2 levels **(C)**.

Figures 6 and 7 show the primary and secondary endpoints analyzed according to the per-protocol principle.

Data on the live birth rate were available in 2 RCTs [21,24]. Metformin increased significantly the live birth rate (OR = 1.94, 95% CI 1.10 to 3.44, *P* = 0.020), without significant heterogeneity across the studies (*P* = 0.230, *I*^2^ = 30%) (Figure 6A).

Based on meta-analysis of all included RCTs [18-24], metformin significantly increased the pregnancy rate (OR = 2.20, 95% CI 1.47 to 3.31, *P* = 0.0001), without significant heterogeneity across the studies (*P* = 0.770, *I*^2^ = 0%) (Figure 6B).

After meta-analysis of 6 RCTs [18-22,24] no significant effect of metformin was observed on the stimulation length (MD = -2.15, 95% CI -5.01 to 0.71, *P* = 0.140), with significant heterogeneity across the studies (*P* < 0.0001, *I*^2^ = 95%) (Figure 7A).

After combining the data from all of the included RCTs [18-24], significantly lower gonadotropin doses were observed under metformin (MD = -282.89, 95% CI -473.23 to -92.55, *P* = 0.040), with significant heterogeneity across the studies (*P* < 0.00001, *I*^2^ = 96.0%; Figure 7B).

The meta-analysis of all included RCTs [18-24] showed a significant effect of metformin on serum E_2_ levels (MD = -190.59, 95% CI -314.52 to -66.67, *P =* 0.003), with significant heterogeneity across the studies (*P* < 0.00001, *I*^2^ = 99.0%) (Figure 7C).

No significant effect of metformin on other secondary endpoints analyzed was detected (data not shown).

## Discussion

The present systematic review and meta-analysis demonstrated that metformin improves the pregnancy rate of more than two-fold with the NNT of 5.7 benefits without heterogeneity among studies [18-24]. This beneficial effect seems to translate into a significantly better rate of live births. In fact, metformin administration increased significantly also the live birth rate of about two-fold. Moreover, only two RCTs [21,24] evaluated the live birth rate as study endpoint on a total of 298 and 363 cycles performed under metformin or no treatment/placebo, respectively.

The benefit of metformin treatment in improving live birth and pregnancy rates was confirmed also after data synthesis performed according to per protocol principle. Although this kind of analysis is generally used for safety-related outcomes, we would explore the specific effect of metformin on gonadotropin stimulation, excluding the patients who had a pregnancy under metformin during the pre-treatment phase [27].

Current data showed a reduced rate of cancelled cycles in PCOS patients who received metformin. In particular, less cycles resulted cancelled both for excessive and poor response under metformin. Of note, in 3 RCTs [21-23] was demonstrated a higher proportion of PCOS patients who achieved monofollicular cycles under metformin, and mono-ovulation should be considered the best result for treating anovulatory infertility.

The reduction in cancellation rate is very important from a clinical point of view, and could be crucial for the reproductive benefit of metformin. However, at the moment, it is not possible to define the real impact of the reduction in cancellation rate on the increased pregnancy/live birth rate. To this regard, a specific data synthesis of the noncancelled cycles would be useful. Unfortunately that analysis was not possible for the lack of this outcome in many papers. Thus, we can hypothesize that the beneficial effect of metformin on reproduction can be explained not only with the fewer cancelled cycles, but also with a potential effect on oocyte quality and/or endometrial competence that cannot be formally excluded.

To this regard, human and animal studies [3-6,28-32] suggested the effect of metformin on the ovary by improving both the intra-ovarian hyperandrogenism and the intra-ovarian insulin-resistance.

Regarding the endometrial receptivity, similarly to what occurred in IVF cycles [8], the current meta-analysis showed that serum E_2_ levels were lower in PCOS patients who received metformin. This figure could affect the endometrial receptivity. However, experimental data [33,34] demonstrated a direct effect of metformin on the endometrium of PCOS patients. Moreover, no effect on the miscarriage rate was detected under metformin, confirming our previous meta-analysis on the lack of any effect of metformin on the risk of miscarriage in infertile PCOS patients under treatment [35]. On the other hand, a reduced risk of miscarriage and of implantation failure under metformin therapy was recently observed in PCOS patients undergoing IVF cycles [8].

Current data demonstrated no beneficial effect of metformin on OHSS risk in patients who received gonadotropins for ovulation induction, whereas only a trend was found for multiple pregnancies. These findings can be explained by the very low risk for and incidence of OHSS and multiple pregnancy observed in many of the included studies due to use of safe gonadotropin protocols and strict criteria for ovulation triggering.

The main strengths of the current systematic review and meta-analysis regard the use of strict inclusion and exclusion criteria, the lack of heterogeneity in all primary reproductive outcomes assessed, and the absence of further clinical trials or RCTs in progress or only designed that make current data conclusive for the next few years.

However, a definitive interpretation of the data and general applicability of the findings to make clinical recommendations seemed to be premature. In fact, the present meta-analysis has several limitations not regarding only the overall study quality (Figure 2).

First of all, it included a limited number of studies and of subjects. In fact, after a careful bibliographic search, only 7 RCTs were included in the final analysis, for an overall population of about 300 subjects. The publication bias was not tested in our analysis in consideration of the low power due to small number of studies included. In addition, many of the included studies had an open design and lacked adequate power analysis.

Secondly, infertile PCOS populations with heterogeneous characteristics were studied. Only 5 out of 7 included RCTs utilized conventional criteria for PCOS diagnosis, no standardization or sub-analysis according to phenotype was possible, and there was no distinction between CC-resistant patients and CC responders. In addition, factors such as insulin resistance and/or body mass index (BMI) may have biased our results. Unfortunately, no sub-analysis according to BMI was possible since almost no included study selected its participants for BMI and only few for insulin resistance. In addition, several confounders related to other patient characteristics, including previous parity, additional infertility diagnosis, duration of infertility, age, and so on may impact our findings.

Finally, protocols for metformin administration were heterogeneous. Specifically, metformin was given with a daily dose of 1500 mg or 1700 mg. These differences in metformin doses could again bias our findings, although recent data [35] demonstrated the lack of significant difference between metformin doses and treatment effectiveness even at lower dosages. On the other hand, metformin was generally administrated as gonadotropin pretreatment and coadministration, and only in 1 RCT [23] it was given as pretreatment. Of note, no study explored the effect of metformin pretreatment alone.

## Conclusions

The current systematic review with meta-analysis demonstrated that metformin administration significantly increases the live birth and pregnancy rates of about two-fold, and reduces the cancellation rate of about 60% in PCOS patients who receive gonadotropins for ovulation induction.

This beneficial effect on reproduction can be explained with an effect on the reduced cancellation rate, even if an effect on oocyte and endometrial quality cannot be excluded.

In consideration of the suboptimal quality of the studies included, further well designed, blinded, placebo-controlled, and adequately powered RCTs are need to confirm current results. Unfortunately, no clinical trial on this issue is currently underway.

## Abbreviations

ASRM: American Society of Reproductive Medicine; BMI: Body mass index; CC: Clomifene citrate; E2: Serum estradiol; ESHRE: European Society of Human Reproduction and Embryology; FSH: Follicle-stimulating hormone; hCG: Human chorionic gonadotropin; HMG: Human menopausal gonadotropins; hpFSH: Highly purified FSH; huFSH: Human urinary FSH; ISI: Institute for scientific information; IVF: In-vitro fertilization; LH: Luteinizing hormone; MD: Mean difference; NIH: National Institute of Health; NNT: Number-needed to treat; OHSS: Ovarian hyperstimulation syndrome; OR: Odds ratio; PCO: Polycystic ovaries; PCOS: Polycystic ovary syndrome; PRISMA: Preferred reporting items for systematic reviews and meta-analyses; RCT: Randomized controlled trial; rFSH: Recombinant FSH.

## Competing interests

The authors declare that they have no competing interests.

## Authors’ contributions

SP and AF made substantial contributions to conception and design, acquisition of data, and analysis and interpretation of data, and to drafting the article. GBLS made substantial contributions to interpretation of data and to revise the study critically. All authors gave final approval of the current version and agreed to be accountable for all aspects of the work in ensuring that questions related to the accuracy or integrity of any part of the work are appropriately investigated and resolved.
